# Role of *Citrullus colocynthis* and *Psidium guajava* Mediated Green Synthesized Silver Nanoparticles in Disease Resistance against *Aeromonas hydrophila* Challenge in *Labeo rohita*

**DOI:** 10.3390/biomedicines11092349

**Published:** 2023-08-23

**Authors:** Ramsha Hafeez, Zakia Kanwal, Muhammad Akram Raza, Shafqat Rasool, Saira Riaz, Shahzad Naseem, Shifa Rabani, Imran Haider, Naushad Ahmad, Suliman Yousef Alomar

**Affiliations:** 1 Department of Zoology, Faculty of Natural Sciences, Lahore College for Women University, Jail Road, Lahore 54000, Pakistan; ramsha.hafeez@lcwu.edu.pk (R.H.); shifa.pu16@gmail.com (S.R.); 2 Centre of Excellence in Solid State Physics, University of the Punjab, Lahore 54590, Pakistan; akramraza.cssp@pu.edu.pk (M.A.R.); shafqatcssp.pu@gmail.com (S.R.); saira.cssp@pu.edu.pk (S.R.); shahzad.cssp@pu.edu.pk (S.N.); 3 Swammerdam Institute for Life Sciences, University of Amsterdam, 1012 Amsterdam, The Netherlands; i.haider@uva.nl; 4 Department of Soil, Plant and Food Sciences, Section of Plant Genetics and Breeding, University of Bari Aldo Moro, 70121 Bari, Italy; 5 Department of Chemistry, College of Science, King Saud University, Riyadh 11451, Saudi Arabia; anaushad@ksu.edu.sa; 6 Zoology Department, College of Science, King Saud University, Riyadh 11451, Saudi Arabia; syalomar@ksu.edu.sa

**Keywords:** phytosynthesis, silver nanoparticles, *Labeo rohita*, *Citrullus colocynthis*, *Psidium guajava*, antibacterial activity

## Abstract

Green synthesis of metallic nanoparticles is an auspicious method of preparing nanoparticles using plant extracts that have lesser toxicity to animal cells and the host. In the present work, we analyzed the antibacterial activity of *Citrullus colocynthis* and *Psidium guajava*-mediated silver nanoparticles (Cc-AgNPs and Pg-AgNPs, respectively) against *Aeromonas hydrophila* (*A. hydrophila*) in an in vivo assay employing *Labeo rohita* (*L. rohita*). *L. rohita* were divided into six groups for both Cc-AgNPs and Pg-AgNPs treatments separately: Control, *A. hydrophila* infected, *A. hydrophila* + Ampicillin, *A. hydrophila* + Cc/Pg-AgNPs (25 µg/L), *A. hydrophila* + Cc/Pg-AgNPs (50 µg/L), and *A. hydrophila* + Cc/Pg-AgNPs (75 µg/L). Changes in different bio-indicators such as hematological, histological, oxidative stress, and cytokine analysis were observed. Interestingly, the infected fish treated with both types of AgNPs (Cc-AgNPs and Pg-AgNPs) exhibited a higher survival rate than the untreated infected fish and demonstrated signs of recovery from the infection, providing a compelling indication of the positive impact of phytosynthesized AgNPs. Disruptions in hematological and histological parameters were found in the infected fish. Both Cc-AgNPs and Pg-AgNPs showed recovery on the hematological and histological parameters. Analysis of oxidative stress and cytokine markers also revealed provoking evidence of the positive impact of Cc-AgNPs and Pg-AgNPs treatment against disease progression in the infected fish. The major finding of the study was that the higher concentrations of the nanoparticles (50 µg/L in the case of Cc-AgNPs and 75 µg/L in the case of Pg-AgNPs) were more effective in fighting against disease. In conclusion, our work presents novel insights for the use of green-synthesized AgNPs as economic and innocuous antibacterial candidates in aquaculture.

## 1. Introduction

A growing range of scientific industries use various technologies to produce engineered nanomaterials, involving various chemical, physical, and biological techniques [1]. Silver (Ag) has long been used in medicine, highlighting its pharmaceutical value [2,3]. Moreover, in recent years, silver nanoparticles (AgNPs) have gained considerable attention for their exciting applications in functionalized surfactants and biocompatible materials. The growing interest in AgNPs reflects the exploration of their potential in the medical field [4,5]. 

*Aeromonas hydrophila* (*A. hydrophila*) is one of the most common fish pathogens that causes serious illnesses in fish. These bacteria frequently cause diseases in freshwater fish, including epizootic ulcerative syndrome, gill rot, tail fin rot, dropsy, septicemia, and ulcers [6]. Furthermore, *A. hydrophila* has developed resistance to several antibiotics, including ampicillin, rifampin, amoxicillin, lincomycin, novobiocin, penicillin, tetracycline, and oxacillin [7]. The rising prevalence of antibiotic resistance has led to an increase in microbial infections and recurrent attacks, necessitating the development of new or modified antimicrobial agents due to the continuous changes in microorganisms through mutation [8]. Therefore, efforts to identify antibiotic alternatives are gaining momentum [7]. 

Through the implementation of green technology, the use of potentially harmful chemicals and solvents has been significantly reduced, leading to the use of non-toxic products and improving material and energy efficiency in various chemical processes. Applying these methods to nanoscience will make it easier to create and work with nanomaterials and nanostructured technologies that are nature-friendly [8]. Synthesizing nanoparticles using biological organisms, such as bacteria and plants, has been proven to be economical, ecologically sustainable, and non-toxic [9,10]. Nanoparticles from plant extracts are now being recognized as potential antibiotic substitutes [11].Plant extracts can be utilized for NPs production on a large scale [12]. Various plant extracts, such as orange peel, *Carica papaya* fruit, *Ziziphus joazeiro* leaf, *Azadirachta indica* leaf, *Ocimum sanctum* leaf, *Stevia* leaf, *Annona reticulata* leaf, *Justicia adhatoda* L. leaf, Citrus lemon leaf, *Petroselinum crispum* leaf, living peanut seedling, and *Coleus aromaticus* leaf, have been utilized as bioreducing agents for the synthesis of nanoparticles. These extracts not only stabilize the nanoparticles but also reduce the metal cations [13,14].

*Citrullus colocynthis* (Cc) leaves are recognized for their antibacterial, antifungal, and anti-inflammatory effects, attributed to the presence of alkaloids and flavonoids in them [15]. *Psidium guajava* (Pg) leaves do not come behind, also have a history of traditional medicinal use, and are known for their antibacterial, anti-inflammatory, and antioxidant properties due to the presence of bioactive compounds such as flavonoids and phenols [16]. 

In the present study, leaf extracts of Cc and Pg were used to prepare *Citrullus colocynthis* silver nanoparticles (Cc-AgNPs) and *Psidium guajava* silver nanoparticles (Pg-AgNPs). The therapeutic potential of both types of nanoparticles was tested for disease treatment in *L. rohita* against *A. hydrophila* infection. *L. rohita* is an economically important fish and is popular for its taste and nutritional significance. Finding new therapeutic measures to control infections in *L. rohita* will hugely benefit the aquaculture industry. We believe that our study presents useful insights for the exploration of novel nanomedicines for combating bacterial infections in aquaculture [17,18,19]. 

## 2. Materials and Methods 

### 2.1. Fish Studies

Healthy fingerlings of *L. rohita* (20 ± 2.0 g, 15 ± 0.5 cm) were obtained from Himalaya Fish Hatchery, Muridke, Pakistan. The fish were transferred randomly to glass aquaria containing 60 L of tap water and equipped with aerators (VENUSAQUA AP-308A, Zhongshan, China). The fish were acclimatized for two weeks before starting the experiment. Pelleted feed (Hi-Tech Aqua Feeds, Pvt. Ltd., Gujranwala, Pakistan) was provided to the fish twice daily at a feeding rate of approximately 3% of their body weight. The 50% water was exchanged daily throughout the experimental period to prevent the deterioration of water quality.

### 2.2. AgNPs Synthesis 

**Figure 1:**
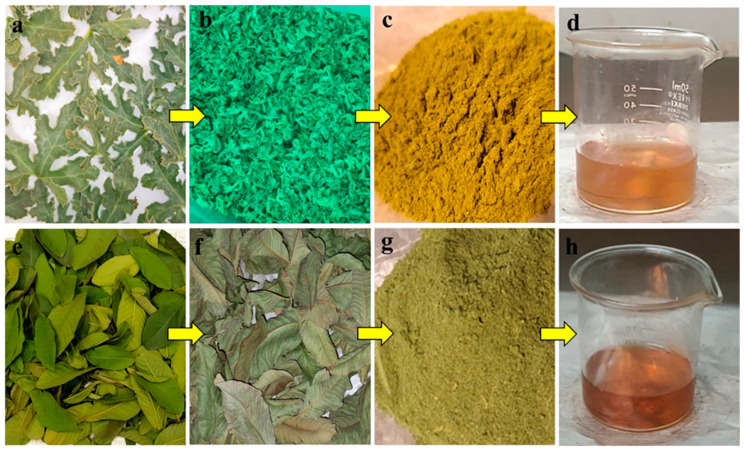
(**a**–**d**) Preparation of *Citrullus colocynthis*-mediated silver nanoparticles (Cc-AgNPs). (**e**–**h**) Preparation of *Psidium guajava*-mediated silver nanoparticles (Pg-AgNPs). (**a**,**e**) Fresh leaves of both plants. (**b**,**f**) Washed and shade dried. (**c**,**g**) Leaves grinded into fine powder. (**d**,**h**) Extract prepared, and color change observed after mixing with AgNO_3_ solution when Cc-AgNPs and Pg-AgNPs formed.

Cc and Pg leaves were collected, washed, and completely shade dried. The dried leaves of Cc and Pg were ground in an electric blender to make a fine powder (Figure 1). A 15 g powder was mixed with 100 mL of ethanol and placed in a Soxhlet apparatus (EAM-9201-06, Seoul, Republic of Korea). It was filtered through Whatman No. 1 filter paper after three hours. The resulting extract was stored at 4 °C for further use. 

In 10 mL of distilled water, 0.2 g of the prepared extract was added and thoroughly mixed before being placed in the rotary apparatus. The solution was filtered using Whatman No. 1 filter paper, and the filtrate was used to reduce silver ions in the AgNO_3_ solution. 1.7 g of AgNO_3_ was mixed with 100 mL of distilled water. 10 mL of this prepared AgNO_3_ solution was mixed with a 4 mL extract solution to synthesize Cc-AgNPs and Pg-AgNPs. After 10 min of continuous stirring and heating at 70 °C, the color of the solution began to change. This color change indicated the formation of AgNPs in the solution (Figure 1d,h).

### 2.3. Characterization of AgNPs

X-ray Diffraction (XRD) (JSX 3201M, Jeol, Tokyo, Japan) was used to determine the crystalline structure of Cc-AgNPs and Pg-AgNPs. The formation of AgNPs was confirmed by measuring UV-Visible spectra (Shimadzu, UV-1800, Kyoto, Japan). Cc-AgNPs and Pg-AgNPs were also examined by Fourier transform infrared (FTIR) spectroscopy (IRTracer-100, Shimadzu, Japan) to determine the attached functional groups. The information about the surface topography and composition of AgNPs was collected using Scanning Electron Microscopy (SEM) (FEI Nova NanoSEM 450, Waltham, MA, USA).

### 2.4. A. Hydrophila Infection Challenge Assay

*A. hydrophila* culture was prepared with approximately 1.3% nutrient broth (HiMedia Kennett Square, PA, USA) dissolved in 100 mL of distilled water. The beakers and test tubes were sterilized in an autoclave at 120 lb. per square inch for 45 min. The bacterial culture was poured into the test tubes and placed in a shaker incubator overnight. The bacterial suspension was confirmed by observing turbidity in the culture test tube, while the blank culture remained clear. The optical density (OD) of the culture was measured using a UV spectrophotometer at a wavelength of 345 nm, and PBS was used to dilute the culture to reach the corresponding concentration of 2 × 10^6^ CFU/mL. Fish were intramuscularly injected with 0.5 mL of the bacterial suspension, and the control group was treated with phosphate-buffered saline (PBS) only. The concentration of AgNPs was kept constant during the whole experimental period, and the experiment was conducted for 10 days. 

#### 2.4.1. Cc-AgNPs Treatment 

Three different concentrations of Cc-AgNPs were given to the fish viz., 25 µg/L, 50 µg/L, and 75 µg/L. Fish were divided into six groups (*n* = 15 per group): Control, *A. hydrophila*, *A. hydrophila* + Ampicillin (75 µg/L), *A. hydrophila* + Cc-AgNPs (25 µg/L), *A. hydrophila* + Cc-AgNPs (50 µg/L), and *A. hydrophila* + Cc-AgNPs (75 µg/L).

#### 2.4.2. Pg-AgNPs Treatment

Three different concentrations of Cc-AgNPs were given to the fish viz., 25 µg/L, 50 µg/L, and 75 µg/L. Fish were divided into six groups (*n* = 15 per group): Control, *A. hydrophila*, *A. hydrophila* + Ampicillin (75 µg/L), *A. hydrophila* + Pg-AgNPs (25 µg/L), *A. hydrophila* + Pg-AgNPs (50 µg/L), and *A. hydrophila* + Pg-AgNPs (75 µg/L). 

### 2.5. Sample Collection 

After the fifth and tenth days post-infection (dpi), fish from all the groups were anesthetized with clove oil (100 µg/L for 40 to 60 s). Blood samples were obtained through cardiovascular punctures for hematological analysis. The gills, liver, and kidney were collected and preserved in 10% formalin for histological analysis. To prepare the liver and gill samples for analysis, they were washed with 9 mL of 0.1 M PBS (pH 7.4). The tissues were then homogenized using a Teflon tissue homogenizer. The homogenate was centrifuged at 4 °C for 10 min at 12,000 rpm, and the supernatant was collected for oxidative stress analysis. Blood was centrifuged at 5000 rpm for 30 min using serum separator gel tubes for cytokine analysis. The resulting tissues and serum supernatant were stored at −20 °C for subsequent analysis.

### 2.6. Hematological Evaluation

Hematological parameters such as Hemoglobin, White Blood Cells (WBCs), Red Blood Cells (RBCs), Mean Corpuscular Volume (MCV), Hematocrit value, Platelets, Mean Corpuscular Hemoglobin (MCH), and Mean Corpuscular Hemoglobin Concentration (MCHC) were analyzed using an automated blood analyzer (Sysmex KX-21, Kobe, Japan). 

### 2.7. Histopathological Evaluation

For histopathological analysis, the organs were dehydrated using graded alcohol solutions. Subsequently, the dehydrated tissues were embedded in paraffin wax and sectioned into thin slices using a microtome. The obtained tissue sections were mounted onto glass slides to enhance cellular visualization and subjected to a series of staining procedures, viz; hematoxylin and eosin staining. Finally, the prepared slides were observed under an optical microscope (Optika B-150DB, Ponteranica, Italy).

### 2.8. Oxidative and Immune Stress Responses

Sodium Dismutase (SOD) (Product Code: A001-1) and Catalase (CAT) (Serial No: A007-1) activities in the tissue homogenate were measured using ELISA kits (Nanjing Jiancheng Bioengineering Institute, Nanjing, China) at a wavelength of 450 nm using the microplate ELISA reader. The Malondialdehyde (MDA) content was measured by a commercial General MDA ELISA kit (Cat: ELK7832) according to the manufacturer’s protocol (ELK Biotechnology Co., Ltd., Denver, CO, USA). Interleukin-1 beta (IL-1β) (Cat: EH0185) was measured by an ELISA kit (Wuhan Fine Biotech. Co., Ltd., Wuhan, China). Tumor Necrosis Factor-alpha (TNF-α) (Cat: ELK1396) was measured by an ELISA kit (ELK Biotechnology Co., Ltd., Denver, CO, USA).

### 2.9. Statistical Analysis

The experimental data was analyzed using GraphPad Prism software (Version 9). ANOVA and Tukey’s post hoc test were performed to analyze group variability. Results with *p* < 0.05 or lower were designated statistically significant (* indicates *p* < 0.05; ** indicates *p* < 0.01; *** indicates *p* < 0.001; **** indicates *p* < 0.0001).

## 3. Results

### 3.1. Analysis of Cc-AgNPs and Pg-AgNPs

#### 3.1.1. XRD Analysis

**Figure 2:**
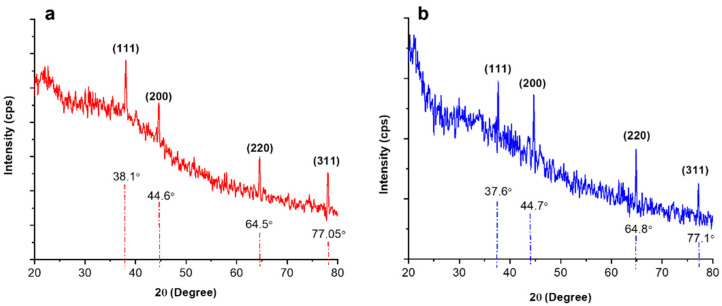
(**a**) XRD patterns of Cc-AgNPs. (**b**) XRD patterns of Pg-AgNPs. Characteristic peaks confirmed the FCC (Face-centered cubic) crystalline structure of AgNPs.

The XRD analysis provided insights about the structural properties and crystallite sizes of Cc-AgNPs and Pg-AgNPs. The crystallite size of both types of AgNPs was determined using the Debye–Scherrer equation [15]. The crystallite size of Cc-AgNPs was determined to be 7 nm, while for Pg-AgNPs, it was found to be 15 nm (Figure 2a,b). The XRD patterns exhibited distinct diffraction planes, confirming the metallic nature of both Cc-AgNPs and Pg-AgNPs. The diffraction patterns also indicated the presence of a face-centered cubic (FCC) crystalline structure in both types of AgNPs, according to COD ID No. 9013046 [15]. 

#### 3.1.2. UV-Vis

**Figure 3:**
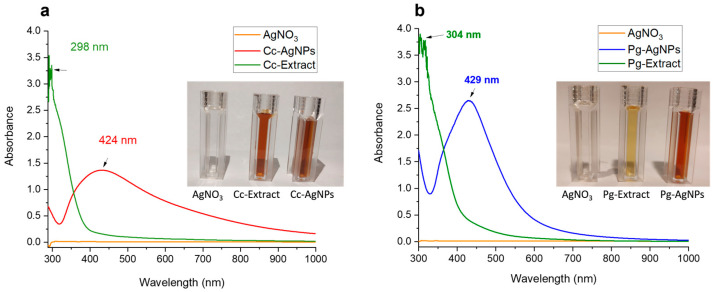
(**a**) UV-Vis spectra of AgNO_3_ solution, Cc-extract, and Cc-AgNPs. (**b**) UV-Vis spectra of AgNO_3_ solution, Pg-extract, and Pg-AgNPs. The inset shows the images of all three colloidal samples.

In Figure 3, the UV-Vis spectra of precursor solution (AgNO_3_), leaf extracts of both plants (Cc-extract and Pg-extract), and AgNPs synthesized using these extracts are presented. No observable peak occurred in the case of the AgNO_3_ solution. The non-appearance of any noticeable peak in the case of precursor solution (AgNO_3_) revealed the absence of any type of AgNPs production without extracting biomolecules. However, some peaks may appear below 300 nm due to silver ions (Ag^+^), as reported in the literature [20]. The noticeable peaks started occurring around 300 nm in both extracts. A relatively broad single Surface Plasmon Resonance (SPR) band appeared around 424 nm for Cc-AgNPs (Figure 3a), while a comparatively sharp SPR band around 429 nm was observed for Pg-AgNPs (Figure 3b). The occurrence of characteristic SPR spectra in the range of 424 nm to 429 nm confirmed the pure silver metallic nature of colloidal nanoparticles (Cc-AgNPs and Pg-AgNPs).

#### 3.1.3. FTIR

**Figure 4:**
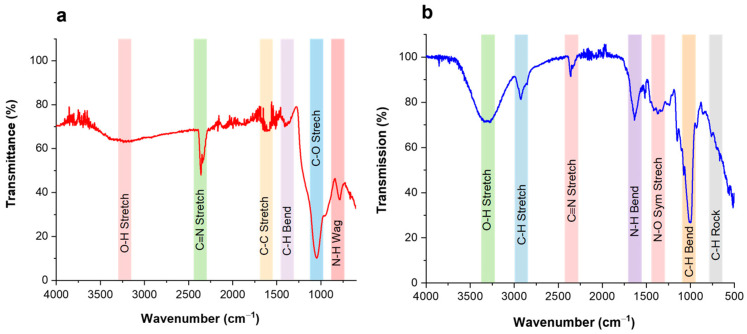
(**a**) FTIR analysis of Cc-AgNPs. (**b**) FTIR analysis of Pg-AgNPs. The occurrence of various absorption bands indicates the existence of extracted biomolecules on the surface of synthesised AgNPs. (Colored strips are a guide to the eye for *x*-axis positions).

The FTIR is a well-known technique for the identification of different functional groups and chemical bonds. The FTIR analysis of green synthesized NPs can be helpful in studying the chemical composition of synthesized NPs. For this purpose, FTIR analysis of Cc-AgNPs and Pg-AgNPs was conducted, and the obtained spectra are presented in Figure 4. The absorption bands of primary significance were observed for Cc-AgNPs at wavenumbers 3240, 2350, 1604, 1403, 1047, and 784 cm^−1^ (Figure 4a). While Pg-AgNPs exhibited absorption bands at wavenumbers 3280, 2929, 2358, 1635, 1374, 1012, and 741 cm^−1^ (Figure 4b). In the case of Cc-AgNPs (Figure 4a), a weak but very broad band occurred around 3240 cm^−1^ that can be due to the presence of an O–H functional group owing to alcoholic or phenolic biomolecules in the Cc-extract. The dagger type band around 2350 cm^−1^ can be assigned to C≡N nitriles, while a weak absorption band appearing around 1604 cm^−1^ may indicate the stretching vibration of C–C aromatic groups. Another weak band around 1403 cm^−1^ can represent the C–H bending vibrations due to alkane. A strong and prominent absorption band around 1047 cm^−1^ might be due to C–O stretching vibrations representing the presence of alcoholic, carboxylic acid, esters, or ether biomolecules. A small band around 784 cm^−1^ can be assigned to N–H waging vibrations due to primary and secondary amines. 

In the case of Pg-AgNPs (Figure 4b), a reasonably strong but broad band around 3280 cm^−1^ can be attributed to the O–H stretching vibrations, signifying the presence of alcoholic, phenolic, and carboxylic acid molecules of Pg-extract on the Pg-AgNPs. The medium absorption band around 2929 cm^−1^ can be designated to the stretching vibration of C–H due to alkaline. The medium-sharp band around 1635 cm^−1^ can be assigned to N–H bending vibration due to 1° amines. The broad medium bands between 1360 and 1290 cm^−1^ can be attributed to N–O symmetry stretching owing to nitro compounds. Similarly, a very strong and sharp band around 1008 cm^−1^ can be assigned to C–H bending vibration because of alkanes. The small bands around 725 cm^−1^ can be assigned to C–H rocking vibrations as a result of alkane molecules [15,21,22].

The FTIR analysis confirmed the manifestation of different functional groups on the surfaces of Cc-AgNPs and Pg-AgNPs. The absorption band signified the existence of extracted biomolecules on the synthesized AgNPs.

#### 3.1.4. SEM

**Figure 5:**
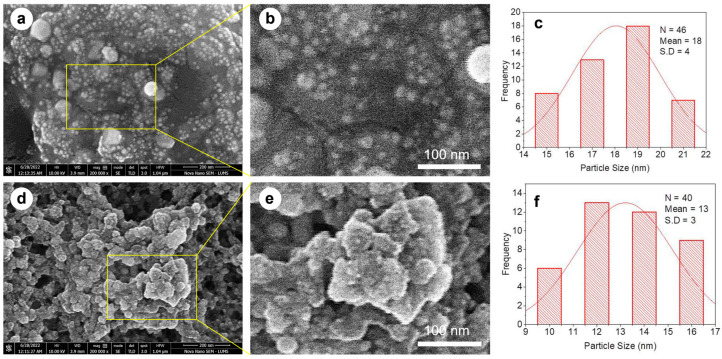
(**a**) SEM analysis of Cc-AgNPs. (**b**) Magnified view of Cc-AgNPs revealing the morphology of individual Cc-AgNPs. (**c**) Cc-AgNPs size distribution histogram. (**d**) SEM analysis of Pg-AgNPs. (**e**) Magnified SEM image of Pg-AgNPs revealing individual Pg-AgNPs (**f**) Pg-AgNPs size distribution histogram. The insect was viewed at ×20,000 magnifications with a 3.9 mm scale (scale bar 100 nm).

The SEM analysis revealed the morphological characteristics of both Cc-AgNPs and Pg-AgNPs (Figure 5). Cc-AgNPs exhibited a pronounced morphology with a round shape and polydispersed distribution, measuring between 15 and 20 nm in size (Figure 5a). Pg-AgNPs displayed a similar morphology, being round and polydispersed, but with a relatively smaller size range of 10–15 nm (Figure 5a). However, in both types of AgNPs, at some places, agglomeration of nanoparticles can be noticed, which results in larger-sized particles (Figure 5b,e). The average size of Cc-AgNPs and Pg-AgNPs, as inferred from the particle size distribution histograms (Figure 5c,f), was found to be 18 ± 4 nm and 13 ± 3 nm, respectively. Considering the limitations of SEM accuracy, the size ranges of both types of AgNPs seem to be virtually comparable.

### 3.2. Behavioral Changes

The behavior of the fish was observed twice daily during both treatments (Table 1). The control group fish exhibited normal behavior (swimming energetically and feeding normally). The *A. hydrophila*-infected fish, on the other hand, moved fast and in an abnormal manner, with less feed intake and irregular fin movement. Their altered feeding behavior showed that they were under stress. In the *A. hydrophila* + Cc-AgNPs (25 µg/L) treatment, the fish was active, swimming, and taking food slowly. The fish infected with *A. hydrophila* + Cc-AgNPs (50 µg/L) were energetic, and their swimming behavior was comparable to that of the control group. The *A. hydrophila* + Cc-AgNPs (75 µg/L) treated group also showed regular swimming and normal feeding behavior. 

When fish were treated with *A. hydrophila* + Pg-AgNPs (25 µg/L), their swimming was normal as compared to the infected fish. *A. hydrophila* + Pg-AgNPs (50 µg/L) fish were observed to show normal swimming and mild hovering behavior. Fish treated with *A. hydrophila* + Pg-AgNPs (75 µg/L) showed improved responses, as indicated by their normal swimming and feeding as compared to the control group. While lower concentrations of both Cc-AgNPs and Pg-AgNPs showed some improvements, the highest concentrations of both types of nanoparticles (50 µg/L and 75 µg/L) appeared to have the most notable positive effect on fish behavior. These findings suggest a dose-dependent relationship, in which higher concentrations may yield more substantial improvements in fish behavior for combating infection.

### 3.3. Hematological Analysis

**Figure 6:**
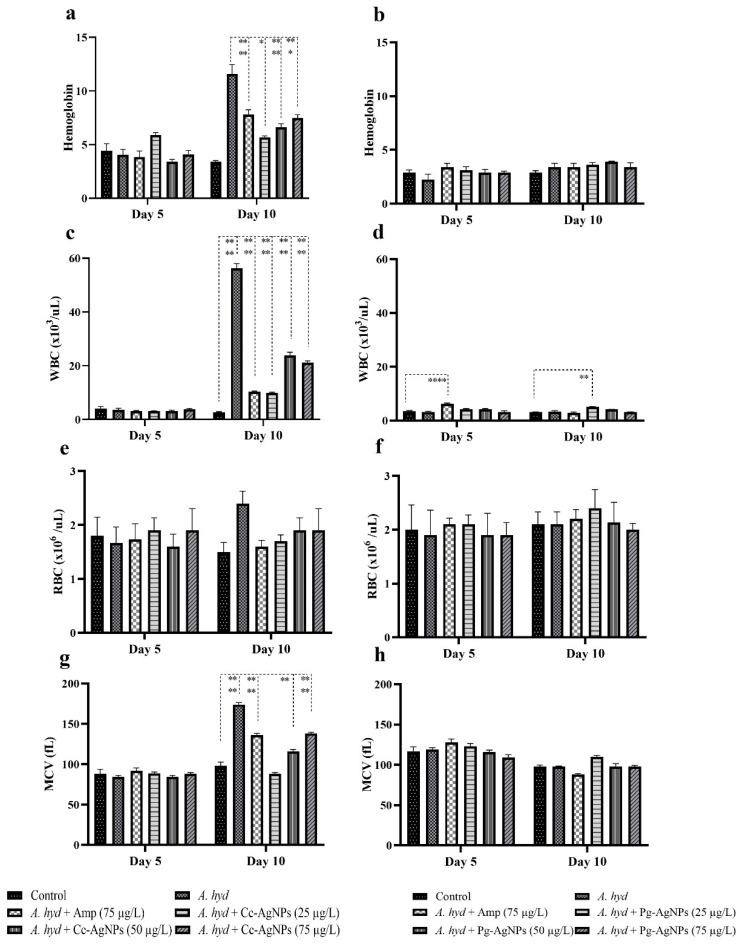
(**a**,**b**) Hemoglobin (Hb); (**c**,**d**) White Blood Cells (WBCs); (**e**,**f**) Red Blood Cells (RBCs); (**g**,**h**) Mean Corpuscular Volume (MCV). * *p* < 0.05; ** *p* < 0.01; **** *p* < 0.0001.

**Figure 7:**
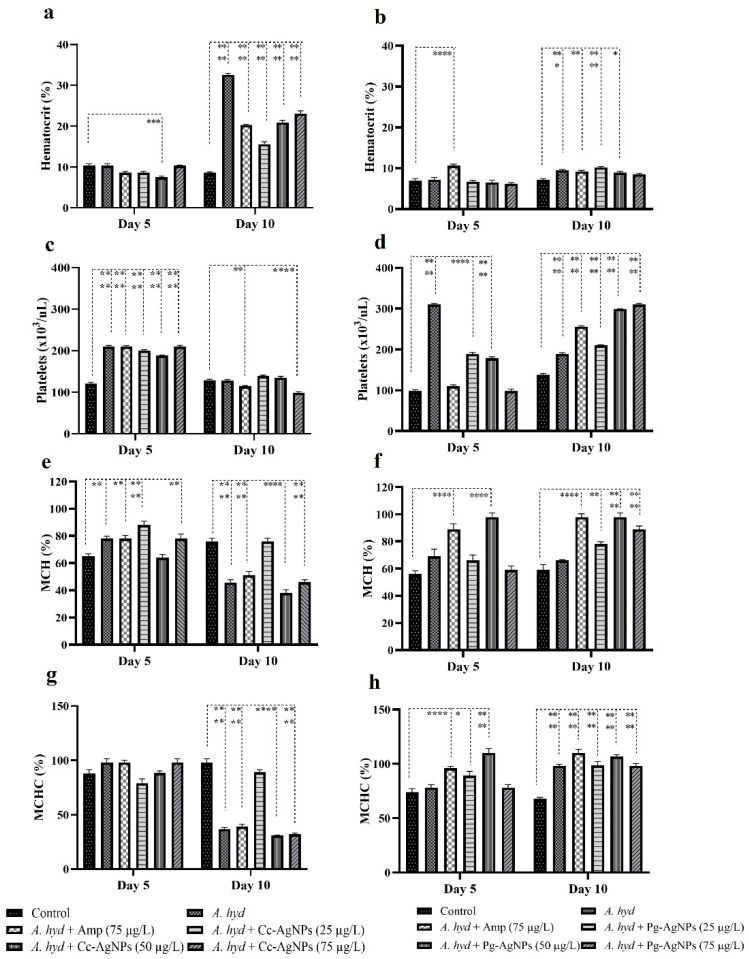
(**a**,**b**) Hematocrit value. (**c**,**d**) Platelets. (**e**,**f**) Mean Corpuscular Hemoglobin (MCH). (**g**,**h**) Mean Corpuscular Hemoglobin Conc. (MCHC). * *p* < 0.05; ** *p* < 0.01; *** *p* < 0.001; **** *p* < 0.0001.

*A. hydrophila* challenge caused a significant increase in platelets and MCH at 5 dpi. However, when treated with the Cc-AgNPs (50 µg/L) MCH% was recovered. In the Pg-AgNPs assay, only platelet numbers were significantly increased in the infected fish, and Pg-AgNPs treatment recovered the platelet number at the highest concentration (75 µg/L). The other blood parameters showed fluctuations in all the infected groups (Figure 6 and Figure 7). 

On 10 dpi in the Cc-AgNPs hemoglobin assay, WBCs, RBCs, MCV, and HCT significantly raised in the infected fish. It was noticed that Cc-AgNPs recovered RBC at all three concentrations, while MCV was recovered by the lowest dose (25 µg/L) of Cc-AgNPs. MCH and MCHC were significantly lower in the infected fish infection as compared to the control. The lowest dose of Cc-AgNPs was again found to restore the normal values of MCH and MCHC. In the Pg-AgNPs assay, HCT, platelets, and MCHC were significantly raised following the challenge. Pg-AgNPs showed a recovery trend in HCT, while other parameters did not show treatment effects (Figure 6 and Figure 7). The hematological parameters also showed variations 1 dpi (Table S1).

### 3.4. Histological Studies

#### 3.4.1. Gills

**Figure 8:**
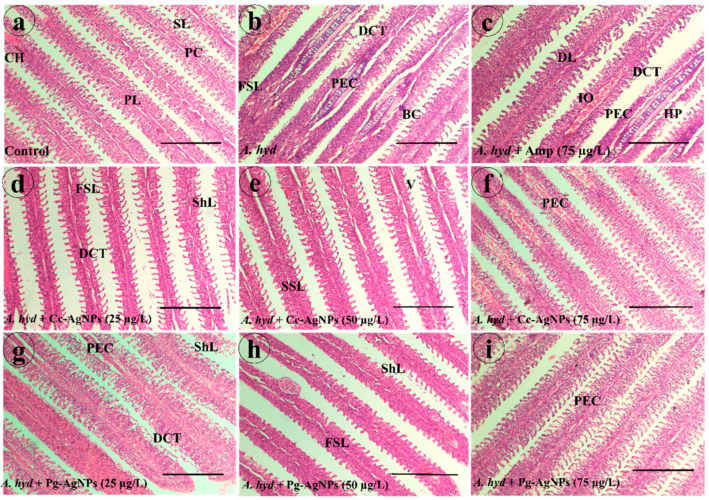
(**a**) Gills of the control group. (**b**) *A. hydrophila*-infected group. (**c**) Ampicillin-treated fish groups. (**d**) *A. hydrophila* + Cc-AgNPs (25 µg/L). (**e**) *A. hydrophila* + Cc-AgNPs (50 µg/L). (**f**) *A. hydrophila* + Cc-AgNPs (75 µg/L). (**g**) *A. hydrophila* + Pg-AgNPs (25 µg/L). (**h**) *A. hydrophila* + Pg-AgNPs (50 µg/L). (**i**) *A. hydrophila* + Pg-AgNPs (75 µg/L). CH—Chloride Cells; PL—Primary Lamellae; SL—Secondary Lamellae; PC—Pavement Cells; ShL—Shortening of lamellae; SSL—Shrinkage of secondary Lamellae; DCT—Dilated club tips; HP—Hyperplasia; FSL—Fusion of secondary lamellae; V—Vacuolation; IO—Interstitial Oedema; DCT—Dilated club tips; PEC—Proliferation of Epithelial cells; BC—Blood congestion; DL—Delocalized Lamellae. Scale bar = (**a**–**i**): 100 µm.

The gills of the control fish showed normal histology with no observable pathological signs. Normal chloride cells, gill filaments, pavement cells, and primary and secondary gill lamellae were present (Figure 8a). Gills of *A. hydrophila*-infected fish showed pathological alterations such as fusion of secondary lamellae, dilated club tips, blood congestion, and proliferation of epithelial cells (Figure 8b). In the ampicillin-treated group, dilated club tips, delocalized lamella, proliferation of epithelial cells, interstitial oedema, and hyperplasia were present (Figure 8c). Gills of *A. hydrophila* + Cc-AgNPs (25 µg/L) showed dilated club tips, shortening of lamellae, and fusion of secondary lamellae (Figure 8d). Fish infected with *A. hydrophila* + Cc-AgNPs (50 µg/L) exhibited some degree of vacuolation and shrinkage of secondary lamella (Figure 8e). Fish infected with *A. hydrophila* + Cc-AgNPs (75 µg/L) showed minor proliferation of epithelial cells (Figure 8f). Fish treated with Pg-AgNPs (25 µg/L) showed proliferation of epithelial cells, shortening of lamellae, and dilated club tips (Figure 8g). *A. hydrophila* + Pg-AgNPs (50 µg/L) group fish showed fusion of secondary lamellae and shortening of lamellae (Figure 8h). *A. hydrophila* + Pg-AgNPs (75 µg/L) group gills showed proliferation of epithelial cells (Figure 8i).

#### 3.4.2. Liver

**Figure 9:**
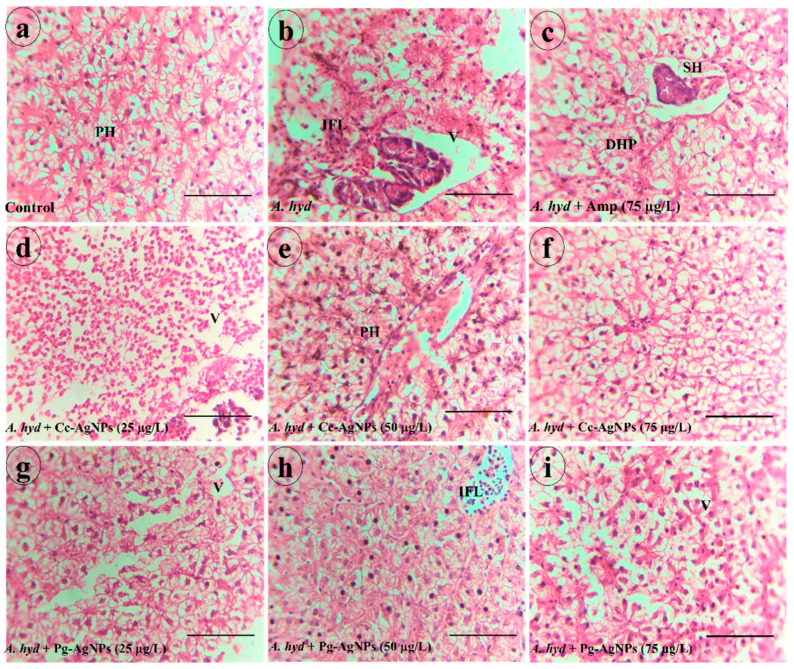
(**a**) Liver of the control group. (**b**) *A. hydrophila* infected group. (**c**) Ampicillin-treated fish groups. (**d**) *A. hydrophila* + Cc-AgNPs (25 µg/L). (**e**) *A. hydrophila* + Cc-AgNPs (50 µg/L). (**f**) *A. hydrophila* + Cc-AgNPs (75 µg/L). (**g**) *A. hydrophila* + Pg-AgNPs (25 µg/L). (**h**) *A. hydrophila* + Pg-AgNPs (50 µg/L). (**i**) *A. hydrophila* + Pg-AgNPs (75 µg/L). PH—Polygonal Hepatocytes; IFL—Infiltration, V—Vacuolation; SH—Swollen Hepatocytes; DHP—Degenerated hepatocyte. Scale bar = (**a**–**i**): 100 µm.

The liver of the control group showed normal hepatocytes with polygonal shapes and hepatic portal veins (Figure 9a). The liver of *A. hydrophila*-infected fish showed different deformities such as infiltration, vacuolation, and a focal area of necrosis (Figure 9b). In the ampicillin-treated group, degenerated hepatocytes, and swollen hepatocytes were seen (Figure 9c). The liver of fish treated with Cc-AgNPs (25 µg/L) showed vacuolation (Figure 9d). Cc-AgNPs (50 µg/L)-treated groups had polygonal hepatocytes and some interstitial spaces (Figure 9e). *A. hydrophila* + AgNPs (75 µg/L) liver showed morphology comparable to the control group (Figure 9f). The livers of fish in the *A. hydrophila* + Pg-AgNPs (25 µg/L) group carried vacuolation (Figure 9g). The group treated with Pg-AgNPs (50 µg/L) had some infiltration and seemingly normal hepatocytes (Figure 9h). *A. hydrophila* + Pg-AgNPs (75 µg/L) in the liver showed vacuolation (Figure 9i). The higher concentration of Cc/Pg-AgNPs proved to be the most effective in reversing the effects of *A. hydrophila* in fish, with the liver showing a similar appearance to the control group.

#### 3.4.3. Kidney

**Figure 10:**
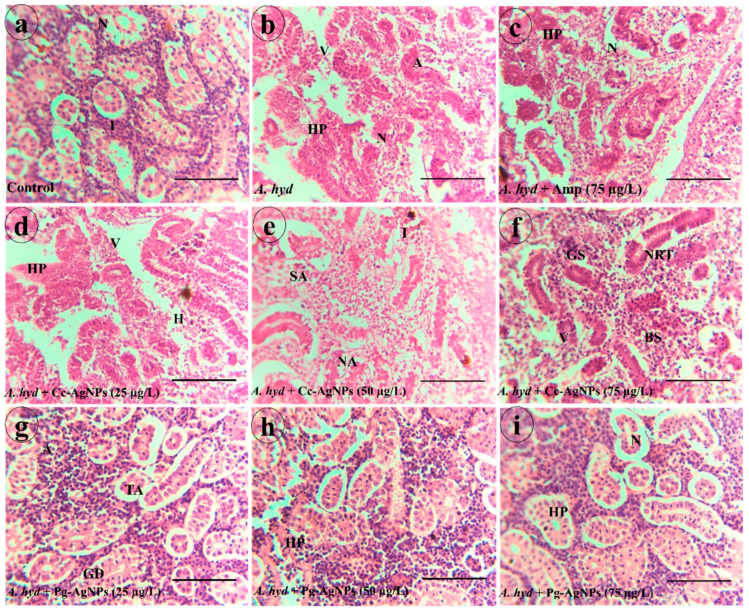
(**a**) Kidney of Control group. (**b**) *A. hydrophila* infected group. (**c**) Ampicillin-treated fish groups. (**d**) *A. hydrophila* + Cc-AgNPs (25 µg/L). (**e**) *A. Hydrophila* + Cc-AgNPs (50 µg/L). (**f**) *A. Hydrophila* + Cc-AgNPs (75 µg/L). (**g**) *A. hydrophila* + Pg-AgNPs (25 µg/L). (**h**) *A. hydrophila* + Pg-AgNPs (50 µg/L). (**i**) *A. hydrophila* + Pg-AgNPs (75 µg/L). G—Glomerulus; HT—hematopoietic tissues; A—Atrophy; BSE—Bowman space enlargement; V—Vacuolization; HP—Hyperplasia; TA—Tubular Alterations; I—Inflammation; SA—Structural Alteration; GS—Glomerulus shrinkage. Scale bar = (**a**–**i**): 100 µm.

The kidney tissue of the control group displayed normal renal tubules with regular glomerulus and hematopoietic tissues (Figure 10a). The kidneys of fish infected with *A. hydrophila* had severe pathological alterations such as necrosis, atrophy, glomerulus shrinkage, vacuolization, and hyperplasia (Figure 10b). In the ampicillin-treated group, necrosis, Bowman space enlargement, vacuolization, glomerulus shrinkage, and hyperplasia were seen (Figure 10c). The kidneys of *A. hydrophila* + Cc-AgNPs (25 µg/L) fish showed vacuolization and hyperplasia (Figure 10d). The *A. hydrophila* + Cc-AgNPs (50 µg/L) group had inflammation and minor structural alterations (Figure 10e). Fish treated with Cc-AgNPs (75 µg/L) showed vacuolization, glomerulus shrinkage, and necrosis (Figure 10f). *A. hydrophila* + Pg-AgNPs (25 µg/L) fish showed inflammation and minor tubular alterations (Figure 10g). The kidneys of fish treated with Pg-AgNPs (50 µg/L and 75 µg/L) showed mild inflammation and restored renal structures (Figure 10h,i).

### 3.5. Antioxidant Analysis

#### 3.5.1. SOD

**Figure 11:**
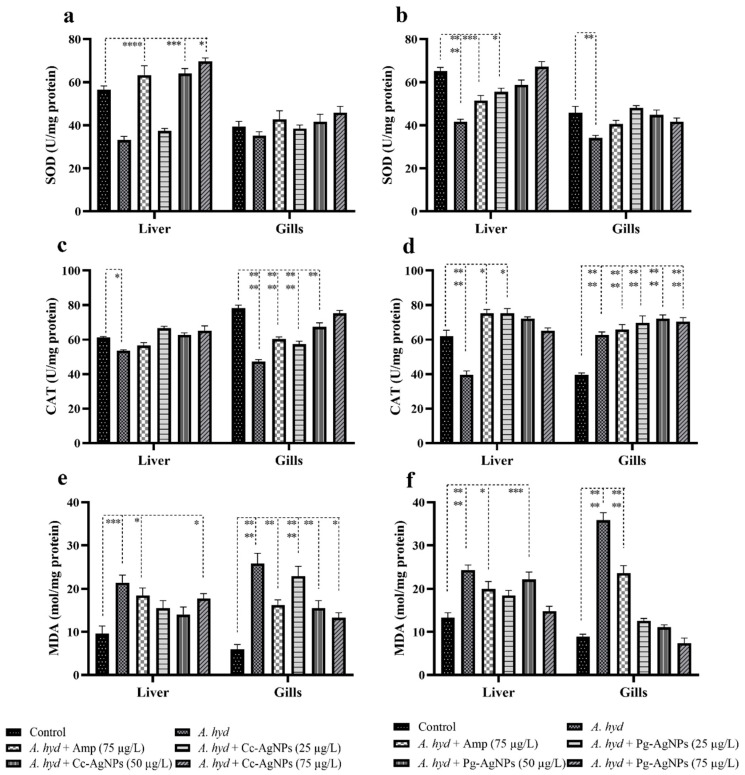
(**a**) Superoxide dismutase (SOD) analysis of Cc-AgNPs treatment. (**b**) SOD analysis of Pg-AgNP treatment. (**c**) Catalase (CAT) activity of Cc-AgNPs treatment. (**d**) CAT activity of Pg-AgNPs treatment. (**e**) Malondialdehyde (MDA) level of Cc-AgNPs treatment. (**f**) MDA level of Pg-AgNPs treatment. Upon Cc/Pg-AgNPs treatment, a significant difference was observed between groups. * *p* < 0.05; ** *p* < 0.01; *** *p* < 0.001; **** *p* < 0.0001.

The SOD was significantly decreased in the liver tissue following *A. hydrophila* challenge in both treatments, viz., Cc-AgNPs and Pg-AgNPs, in comparison to the control group (Figure 11a,b). The level of SOD was recovered by both Cc-AgNPs and Pg-AgNPs at all concentrations (25, 50, and 75 µg/L). The SOD was also significantly decreased in the infected fish gills; both Ampicillin and Pg-AgNPs (at all three concentrations) recovered the normal levels of SOD in the gills (Figure 11b).

#### 3.5.2. CAT

A significant decrease in CAT activity was observed in the infected group’s liver as compared to the control (Figure 11c,d). Both Cc-AgNPs and Pg-AgNPs, recovered CAT levels at all concentrations. Ampicillin also recovered the CAT levels. (Figure 11c,d). CAT was also decreased significantly in the gills (Figure 11c), which was recovered by Cc-AgNPs at the highest concentration (75 µg/L) (Figure 11c). No significant variations in the CAT level in gills were observed in the case of the Pg-AgNPs assay (Figure 11d).

#### 3.5.3. MDA

Interestingly, the MDA levels were significantly enhanced in the infected fish liver (Figure 11e,f). In the case of Cc-AgNPs, 25 and 50 concentrations recovered the MDA levels, while in the case of Pg-AgNPs, 25 and 75 concentrations restored the normal level of MDA. Gills also showed a significant increase in MDA following infection (Figure 11e,f). Only Pg-AgNPs (with all three concentrations) recovered the normal values of MDA (Figure 11f).

### 3.6. Cytokines Analysis

**Figure 12:**
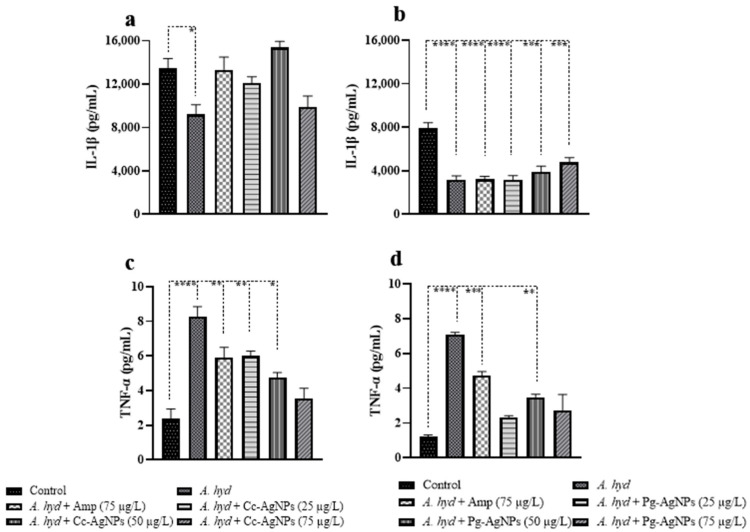
(**a**) Interleukin 1 beta (IL-1β) expression of Cc-AgNPs treatment (**b**) IL-1β expression of Pg-AgNPs treatment. (**c**) Tumor necrosis factor alpha (TNF-α) level in serum of Cc-AgNPs treatment. (**d**) TNF-α level in serum of Pg-AgNPs treatment. * *p* < 0.05; ** *p* < 0.01; *** *p* < 0.001; **** *p* < 0.0001.

IL-1β was significantly downregulated in the infected fish (Figure 12a,b). Ampicillin and Cc-AgNPs at all three concentrations recovered the IL-1β levels. Pg-AgNPs also showed a recovery trend in the increasing values of IL-1β in the Pg-AgNP-treated groups (Figure 12b). On the other hand, TNF-α increased significantly after challenge (Figure 12c,d). Cc-AgNPs (at concentrations 75 µg/L) and Pg-AgNPs (at concentrations 25 µg/L and 75 µg/L) effectively recovered the TNF-α levels (Figure 12c,d).

## 4. Discussion

Using phytosynthesized nanoparticles is a sustainable and environmentally friendly way to treat diseases without using chemicals or antimicrobial drugs. In addition to being harmful to the environment and people’s health, these chemicals also promote the growth of antibiotic-resistant bacterial strains and weaken the immune system. As a result, developing other strategies to combat bacterial challenges is necessary [23]. The antibacterial activities of AgNPs have previously been demonstrated to be effective against *Escherichia coli* [24]. In the current study, the phytoreduction approach was employed to synthesize AgNPs using the leaf extracts of *Citrullus colocynthis* and *Psidium guajava*. Both extracts served as reducing as well as stabilizing agents, while silver nitrate was used as a precursor. As the precursor dissolved into the water, it produced silver ions (Ag^+^), the introduction of extract biomolecules into the solution under optimized experimental conditions provided the required electrons to complete “bioreduction” process. The free silver atoms (Ag^0^) produced the silver nuclei under van der Waals interactions and Brownian motion. During the nucleation and growth processes, AgNPs synthesis reaction is accomplished. In addition to the reducing and stabilizing agents, the extracted biomolecules may deposit on the surface of AgNPs to enhance their effectiveness in various applications.

The characterization results of XRD, UV-Vis, FTIR, and SEM techniques confirmed the successful formation of Cc-AgNPs and Pg-AgNPs. The XRD analysis demonstrated the crystalline nature of both types of silver nanoparticles. Furthermore, XRD analysis also indicated a slight variation in the crystallite size of both types of AgNPs. The UV-Vis spectra revealed characteristic absorption peaks at 424 nm for Cc-AgNPs and 429 nm for Pg-AgNPs, indicative of their pure silver nature. The broadness of the SPR band can be related to the size distribution of metallic NPs. The broader SPR band of Cc-AgNPs in comparison with that of Pg-AgNPs indicated that Cc-AgNPs are relatively larger in size and have a wider size distribution, while Pg-AgNPs are comparatively smaller in size and are more uniform in their size distribution. The SEM morphological analysis of both types of AgNPs again confirmed the size variation. However, as mentioned above, this difference in size between Cc-AgNPs and Pg-AgNPs can be considered virtually similar. So, we believe that the biological activity of green synthesized AgNPs can be attributed to the synergistic effect of silver NPs and the presence of extract biomolecules. Further studies are recommended for precise investigations in this regard.

These bioactive substances also acted as stabilizing/capping agents and catalysts for the redox processes [25,26]. Through synergistic effects, the presence of these phytochemicals on nanoparticle surfaces may improve the efficacy of AgNPs as antibacterial agents. In the precursor solution (AgNO_3_), no noticeable peak can be seen in the visible region (about 400–700 nm). However, some peaks may appear below 300 nm due to silver ions (Ag^+^), as reported in the literature [20]. The AgNPs hydrogel that has been developed exhibits promising potential as a secure wound treatment modality, with the ability to effectively eradicate infections and offer a safe and efficacious approach to the management of infected wounds [27].

As blood responds to internal and external environmental changes, hematological parameters help diagnose diseases and determine how healthy an organism is [28]. Our investigation revealed noteworthy positive changes on the hematological parameters following the administration of Cc-AgNPs and Pg-AgNPs in infected fish. These findings indicate that both types of nanoparticles, at similar doses, may have differential effects on the hematological indices of *A. hydrophila*-infected fish. Cc-AgNPs exhibited a more pronounced influence on specific blood parameters, while Pg-AgNPs demonstrated a distinct impact on others. The observed variations may be attributed to the unique physicochemical properties and composition of each type of nanoparticle. Moreover, the recovery of blood parameters by the Cc-AgNPs and Pg-AgNPs indicated that they may enhance the fish’s immune response, enabling them to better withstand the bacterial challenge. The NPs made with biological extracts exhibit a stronger antibacterial impact than those made through chemical reduction [29]. AgNPs synthesized via biosynthesis utilizing *Aspergillus niger* were observed in relation to *Escherichia coli*, wherein a complete disruption of the bacterial membrane was observed after contact [30].

Histopathological examinations of fish tissues may offer valuable insights into the response to nanoparticle treatments. *A. hydrophila*-infected fish showed significant pathological alterations in the gills, liver, and kidney. Treatment with both Cc-AgNPs and Pg-AgNPs ameliorated some of these effects, particularly at higher doses. It has been found previously that silver nanoparticles exhibit effective bactericidal activity within the concentration range of 62–250 μg/mL while demonstrating fungicidal activity at higher concentrations of 125–500 μg/mL [31]. The antibacterial and antioxidant properties of the green-generated AgNPs from the *Terminalia chebula* fruit on zebrafish embryos further suggest their potential as medicinal agents [32]. In the present study, the kidney of *L. rohita* infected with *A. hydrophila* exhibited glomerulus shrinkage, hyperplasia, vacuolization, and inflammation. However, when treated with Cc/Pg-AgNPs at different concentrations, these symptoms were observed to be mitigated and recovered. *Rhamnus triquetra* leaves were used as a stabilizing agent in the formation of nickel oxide nanoparticles, which exhibited antimicrobial efficacy against various bacterial and fungal strains and had a recovery effect [33].

The antitumor activity of AgNPs in animals has been demonstrated to be a result of their capacity to obstruct the function of abnormally expressed signaling proteins [34]. Antioxidants are considered necessary components that prevent or lessen free radical reactions, prevent or stop cellular damage, and play a crucial role in preventing oxidative stress in organ damage [35]. In antioxidant systems, SOD and CAT serve as the initial lines of defense [36]. Superoxide anion radical dismutation into water and hydrogen peroxide, which catalyzes and reduces to water and oxygen for the elimination of ROS, is the primary activity of the SOD enzyme [37]. In the present study, a significant decrease in SOD activity was observed in the gill and liver tissues of the infected group compared to the control. However, treatment with Cc/Pg-AgNPs resulted in an increase in SOD activity, indicating a potential role in increasing ROS scavenging and reducing ROS accumulation, thereby mitigating tissue damage. With 50 μg/L of the AgNPs solution, silver nanoparticles bio-synthesised with plant extracts such as *Ocimum tenuiflorum*, *Citrus sinensis*, and *Syzygium cumini* had a growth inhibition zone between 12 and 17 mm when used against *Staphylococcus* aureus [38].

After 10 dpi, a significant decrease in CAT activity in the infected group as compared to the control group was seen, which recovered with Cc/Pg-AgNPs treatment in their gill and liver tissue. This improvement in antioxidant defense may be based on increased oxygen-free radical production, encouraging antioxidant activities to shield cells from free radical damage [39]. *Cyprinus carpio* treatment with AgNPs showed variations in antioxidant enzyme activity [40]. The superoxide radical may have been inactivated because of copper exposure, which caused the CAT activity in the gill to decrease significantly. Until day two, the CAT activity of diverse tissues treated with 50 mg/L AgNPs showed a slight decline, and after that, a significant increase was seen [41].

Oxidative stress is a common cellular damage mechanism in tissues present after challenge with *A. hydrophila*. One early response released by macrophages and monocytes and circulating in the blood is IL-1β. IL-1β expression was observed to increase after treatment with Cc-AgNPs and Pg-AgNPs, recovering the effect of *A. hydrophila* infection. According to a different study, *Moringa oleifera* leaf extracts significantly reduced the generation of nitric oxide and other inflammatory markers like interleukin-6 (IL-6) and IL-1β [42]. MDA contents in the gill and liver tissues of *L. rohita* subjected to infection were observed to be significantly increased compared to the control. However, following the tenth day of exposure to 25, 50, and 75 µg/L of Cc/Pg-AgNPs, a significant decrease in MDA content in the gills and liver was observed, restoring the effect of infection, which increased after infection.

All three concentrations of 25 µg/L, 50 µg/L, and 75 µg/L AgNPs showed positive effects for infection clearance; however, the highest recovery was seen at 50 and 75 µg/L. It is noteworthy that as an internal control, we also administered solely Cc-AgNPs and Pg-AgNPs (with their highest concentrations used in the study: 75 µg/L) to uninfected fish to check their effects on the physiological markers. However, we did not find any significant differences between the control and sole Cc-AgNPs and Pg-AgNPs treated groups on any parameter described in this study, thereby indicating their safe administration to the fish. The antibacterial efficacy of AgNPs has been observed to be dose-dependent and to exhibit a higher potency against Gram-negative bacteria in comparison to Gram-positive bacteria [43]. Infections in aquatic animals caused by *A. hydrophila* can be treated with phytosynthesized AgNPs, which are cheaper and better than antibiotics and other biocides [44]. Therefore, we suggest that phytosynthesized Cc-AgNPs at our high concentration and Pg-AgNPs at our medium concentration caused a decrease in fish infection by enhancing the immune status of fish. The effect is more on the adaptive part of the immune system. The higher concentration of Cc/Pg-AgNPs proved to be the most effective in reversing the effects of *A. hydrophila* in fish, with the liver showing a similar appearance to the control group.

## 5. Conclusions

In the present study, we investigated the therapeutic potential of phytosynthesized Cc-AgNPs and Pg-AgNPs against *A. hydrophila* infection in *L. rohita*. Our results demonstrated that Cc-AgNPs and Pg-AgNPs show recovery in hematology, histology, antioxidant enzyme activity, and cytokine activity in the infected fish. Moreover, our results highlight the dose-dependent response of the AgNP effect. Higher concentrations of Cc-AgNPs (75 µg/L) and medium concentrations of Pg-AgNPs (50 µg/L) exhibited the best efficacy against infection. The use of Cc-AgNPs and Pg-AgNPs as alternatives to antibiotics in the aquaculture industry hold great promise. Future research should be conducted to explore the efficacy of other biologically synthesized AgNPs for developing novel bacterial infection treatments through green nanomedicines.

## Figures and Tables

**Table 1:** Behavioral attributes recorded after *A. hydrophila* challenge in both Cc-AgNPs and Pg-AgNPs treatments. Normal (+), Severe (−), Mild (+−).

Groups	Swimming	Feed Intake	Fin Movement	Hovering Behavior
Control	+	+	+	+
*A. hyd*	−	−	+−	−
*A. hyd* + Amp (75 µg/L)	−	+	+−	+
*A. hyd* + Cc-AgNPs (25 µg/L)	+	+−	+	+
*A. hyd* + Cc-AgNPs (50 µg/L)	+	+	+	+
*A. hyd* + Cc-AgNPs (75 µg/L)	+−	+−	+−	+
*A. hyd* + Pg-AgNPs (25 µg/L)	+	+−	+	+
*A. hyd* + Pg-AgNPs (50 µg/L)	+	+	−	+−
*A. hyd* + Pg-AgNPs (75 µg/L)	+	+	+−	+

## Data Availability

Data will be available on request from the corresponding author.

## Supplementary Materials

The following supporting information can be downloaded at: https://www.mdpi.com/article/10.3390/biomedicines11092349/s1, Table S1: Summary of hematological parameters at 1 dpi. Values with a different superscript in the same row are significantly different.
