# Supplementary material for: Role of Citrullus colocynthis and Psidium guajava Mediated Green Synthesized Silver Nanoparticles in Disease Resistance against Aeromonas hydrophila Challenge in Labeo rohita
Source: Biomedicines. 2023 Aug 23;11(9):2349. doi: 10.3390/biomedicines11092349 (PMC10525728; doi:10.3390/biomedicines11092349)
Supplement: Supplementary file 1 [file biomedicines-11-02349-s001.zip › biomedicines-2500355-supplementary.pdf]

Table S1. Summary of hematological parameters at 1 dpi. Values with a different superscript in the same row are significantly different.

Hematological indices		Control	*A. hyd*	*A. hyd* + Amp (75 µg/L)	*A. hyd* + AgNPs (25 µg/L)	*A. hyd* + AgNPs (50 µg/L)	*A. hyd* + AgNPs (75 µg/L)
Cc-AgNPs - Day 1	Hemoglobin g/dl	3.60 ± 0.23[a]	3.40 ± 0.11[a]	3.30 ± 0.28[a]	3.10 ± 0.34[a]	4.60 ± 0.23[a]	3.30 ± 0.11[a]
	WBC (TLC) x10³ /uL	2.10 ± 0.34[a]	3.20 ± 0.40[a]	2.30 ± 0.46[a]	4.90 ± 0.23[b]	6.60 ± 0.23[c]	2.90 ± 0.28[a]
	Total RBC x10⁶ /uL	1.80 ± 0.34[a]	1.60 ± 0.28[a]	16.0 ± 3.46[b]	2.10 ± 0.34[a]	1.90 ± 0.40[a]	1.50 ± 0.17[a]
	MCV fL	75.00 ± 1.73[a]	95.00 ± 1.73[b]	92.10 ± 4.04[b]	89.00 ± 5.19[b]	88.00 ± 1.73[b]	89.00 ± 1.73[b]
	HCT (PVC) %	6.30 ± 0.46[a]	6.60 ± 0.23[a]	8.93 ± 0.26[b]	11.30 ± 0.28[c]	12.27 ± 0.43[c]	8.60 ± 0.23[a]
	Platelets x10³ /uL	232.00 ± 4.04[a]	345.00 ± 2.88[b]	455.00 ± 2.88[c]	211.00 ± 1.73[a]	110.00 ± 2.30[d]	256.00 ± 1.15[a]
	MCH %	89.00 ± 2.30[a]	89.00 ± 1.73[a]	58.00 ± 1.73[b]	69.00 ± 3.46[b]	89.00 ± 4.04[a]	69.00 ± 2.30[a]
	MCHC %	100.00 ± 4.61[a]	110.00 ± 2.88[b]	66.00 ± 2.30[c]	89.33 ± 1.85[d]	98.00 ± 3.46[a]	88.00 ± 4.61[d]
Pg-AgNPs- Day 1	Hemoglobin g/dl	3.40 ± 0.11[a]	3.60 ± 0.23[a]	3.40 ± 0.34[a]	3.76 ± 0.92[a]	3.80 ± 0.34[a]	3.20 ± 0.17[a]
	WBC (TLC) x10³ /uL	3.90 ± 0.57[a]	2.70 ± 0.11[a]	4.50 ± 0.28[a]	4.60 ± 0.23[a]	3.60 ± 0.23[a]	3.40 ± 0.34[a]
	Total RBC x10⁶ /uL	1.90 ± 0.40[a]	1.80 ± 0.40[a]	2.10 ± 0.11[a]	2.50 ± 0.17[a]	2.10 ± 0.17[a]	2.10 ± 0.11[a]
	MCV fL	110.00 ± 1.15[a]	119.00 ± 2.30[a]	99.00 ± 2.30[a]	95.00 ± 1.73[a]	118.00 ± 3.46[a]	110.00 ± 1.15[a]
	HCT (PVC) %	8.40 ± 0.23[a]	6.80 ± 0.34[b]	6.20 ± 0.11[b]	6.10 ± 0.34[b]	6.60 ± 0.23[b]	7.20 ± 0.23[a]
	Platelets x10³ /uL	218.00 ± 3.46[a]	118.00 ± 2.30[b]	221.00 ± 1.73[a]	98.00 ± 3.46[b]	122.00 ± 1.15[b]	188.00 ± 2.30[a]
	MCH %	56.00 ± 2.30[a]	59.00 ± 1.73[a]	56.00 ± 1.15[a]	59.00 ± 2.30[a]	65.67 ± 1.45[a]	65.67 ± 2.02[a]
	MCHC %	49.00 ± 2.30[a]	67.00 ± 2.88[b]	68.00 ± 3.46[b]	74.00 ± 2.30[b]	75.00 ± 1.15[b]	98.00 ± 1.15[b]
